# Allogeneic MHC-matched T-cell receptor α/β-depleted bone marrow transplants in SHIV-infected, ART-suppressed Mauritian cynomolgus macaques

**DOI:** 10.1038/s41598-022-16306-z

**Published:** 2022-07-19

**Authors:** Jason T. Weinfurter, Saritha S. D’Souza, Lea M. Matschke, Sarah Bennett, Laurel E. Kelnhofer-Millevolte, Kran Suknuntha, Akhilesh Kumar, Jennifer Coonen, Christian M. Capitini, Peiman Hematti, Thaddeus G. Golos, Igor I. Slukvin, Matthew R. Reynolds

**Affiliations:** 1 Department of Pathobiological Sciences, School of Veterinary Medicine, University of Wisconsin, Madison, WI 53706 USA; 2 Wisconsin National Primate Research Center, University of Wisconsin, Madison, WI 53715 USA; 3 Present Address: Chakri Naruebodindra Medical Institute, Faculty of Medicine Ramathibodi Hospital, Mahidol University, Samut Prakan, Thailand; 4 Department of Pediatrics, School of Medicine and Public Health, University of Wisconsin, Madison, WI 53792 USA; 5 Carbone Cancer Center, School of Medicine and Public Health, University of Wisconsin, Madison, WI 53792 USA; 6 Division of Hematology/Oncology/Bone Marrow Transplantation, Department of Medicine, University of Wisconsin Hospital and Clinics, Madison, WI 53792 USA; 7 Department of Comparative Biosciences, School of Veterinary Medicine, University of Wisconsin, Madison, WI 53706 USA; 8 Department of Obstetrics and Gynecology, University of Wisconsin-Madison, Madison, WI 53792 USA; 9 Department of Pathology and Laboratory Medicine, School of Medicine and Public Health, University of Wisconsin, Madison, WI 53705 USA; 10 Department of Cell and Regenerative Biology, University of Wisconsin School of Medicine and Public Health, Madison, WI USA

**Keywords:** Bone marrow transplantation, HIV infections

## Abstract

Allogeneic hematopoietic stem cell transplants (allo-HSCTs) dramatically reduce HIV reservoirs in antiretroviral therapy (ART) suppressed individuals. However, the mechanism(s) responsible for these post-transplant viral reservoir declines are not fully understood. Therefore, we modeled allo-HSCT in ART-suppressed simian-human immunodeficiency virus (SHIV)-infected Mauritian cynomolgus macaques (MCMs) to illuminate factors contributing to transplant-induced viral reservoir decay. Thus, we infected four MCMs with CCR5-tropic SHIV162P3 and started them on ART 6–16 weeks post-infection (p.i.), maintaining continuous ART during myeloablative conditioning. To prevent graft-versus-host disease (GvHD), we transplanted allogeneic MHC-matched α/β T cell-depleted bone marrow cells and prophylactically treated the MCMs with cyclophosphamide and tacrolimus. The transplants produced ~ 85% whole blood donor chimerism without causing high-grade GvHD. Consequently, three MCMs had undetectable SHIV DNA in their blood post-transplant. However, SHIV-harboring cells persisted in various tissues, with detectable viral DNA in lymph nodes and tissues between 38 and 62 days post-transplant. Further, removing one MCM from ART at 63 days post-transplant resulted in SHIV rapidly rebounding within 7 days of treatment withdrawal. In conclusion, transplanting SHIV-infected MCMs with allogeneic MHC-matched α/β T cell-depleted bone marrow cells prevented high-grade GvHD and decreased SHIV-harboring cells in the blood post-transplant but did not eliminate viral reservoirs in tissues.

Allogeneic hematopoietic stem cell transplants (allo-HSCTs) are essential curative therapies for high-risk hematologic malignancies^1–3^, replacing recipient cells of hematopoietic origin with donor cells. In persons with HIV, allo-HSCTs also dramatically reduce viral reservoirs, enabling several transplant recipients to prevent or delay viral rebound after stopping antiretroviral therapy (ART)^4–10^. While allo-HSCTs are potent cellular immunotherapies, they are not scalable nor relevant to most people living with HIV without underlying hematologic cancers. Therefore, understanding the mechanism(s) of allo-HSCT-mediated reservoir decay can aid in designing safer and more efficacious treatment regimens. To this end, allo-HSCT components postulated to reduce viral reservoirs include pre-transplant conditioning regimens, protecting donor CD4^+^ T cells from infection with ART, graft-versus-host (GvH) responses, and donor HSCs lacking functional C-C chemokine receptor type 5 (CCR5)^11,12^.

To enter cells, HIV must sequentially bind to CD4 and a cellular coreceptor, primarily chemokine receptors CCR5 and CXCR4. These molecular interactions are disrupted in individuals homozygous for the *CCR5∆32* gene variant (*∆32*/*∆32*), which prevents functional CCR5 from being expressed on cell surfaces, making *∆32*/*∆32* cells resistant to infection with CCR5-tropic HIV strains. The protective properties of *∆32*/*∆32* cells were employed in the hematologic cancer treatments of two men with HIV. In these men, transplanting *∆32*/*∆32* HSCs induced cancer remission and the rapid depletion of HIV reservoirs, allowing the “Berlin” and “London” patients to stop ART for at least 4 years without viral rebound^5,6,8,13^. Undoubtedly, the absence of CCR5 on donor cells prevented virus replication and the reestablishment of viral reservoirs post-transplant, supporting long-term HIV remission. However, additional transplant-related factors must have cleared pre-existing viral reservoirs.

Transplants with *∆32* heterozygous or CCR5 wild-type donors in ART-suppressed recipients provide further insights into eliminating latent HIV^4,7,9,14,15^. Notably, three CCR5 wild-type HSC recipients substantially reduced their viral reservoirs post-transplant and delayed HIV rebound up to 9 months after analytical treatment interruption (ATI)^4,15^. Mathematical modeling suggests that only hundreds to thousands of latently infected cells remained in these individuals at ART withdrawal^16^. Assuredly, maintaining ART during the peri-transplant period was an essential component of the allo-HSCT regimens, limiting HIV's spread from recipient cells to vulnerable donor cells^17^. Importantly, these allo-HSCTs also indicate that factors outside of donor genetics contribute to depleting viral reservoirs.

An attractive explanation for allo-HSCT-induced reservoir decay is the pre-transplant conditioning regimens used to reduce malignant cell burdens, create space for HSC engraftment, and ablate immune cells mediating graft rejections^11,18^. However, studies in humans and nonhuman primates (NHPs) indicate that pre-transplant conditioning regimens only transiently reduce viral reservoirs^19–24^, which are refilled by the homeostatic or antigen-driven proliferation of latently infected cells^17,22^. Thus, cytoreductive treatments alone are insufficient for eradicating HIV reservoirs, even when ART is maintained.

A more likely mechanism for allo-HSCT-mediated viral reservoir decay is the beneficial effects of GvH responses^7,15,25^. After allo-HSCTs, major histocompatibility complex (MHC)-matched donor T cells recognize minor alloantigens expressed by host leukocytes, eliminating malignant and HIV-infected cells simultaneously, thereby mediating graft-versus-tumor and graft-versus-viral reservoir (GvVR) effects, respectively^26,27^. But these GvH interactions can also result in life-threatening graft-versus-host disease (GvHD), a condition where donor cells attack non-hematopoietic origin cells or tissues. The primary cells mediating GvHD post-transplant are canonical T cells expressing αβ T cell receptors^28,29^, prompting the development of allo-HSCT regimens using cell preparations selectively depleted of αβ T cells^30,31^. Disentangling allo-HSCT's GvVR effects from GvHD may improve the safety and efficacy of cellular therapies targeting viral reservoirs.

In humans, variability in treatment regimens makes it challenging to investigate allo-HSCTs effects on HIV reservoirs. However, allo-HSCT treatment conditions can be standardized in NHP models of HIV infection. To this end, cynomolgus macaques (*Macaca fascicularis*) from the island of Mauritius are well-suited to model allo-HSCTs in people with HIV. Mauritian cynomolgus macaques (MCM) are descended from a small founder population^32^, resulting in limited genetic diversity, even at polymorphic loci. As a result, MCMs have only seven MHC haplotypes (termed M1-M7), facilitating the assembly of MHC-matched transplant pairs^33–35^. Moreover, SIV and simian-human immunodeficiency viruses (SHIVs) infections in MCMs recapitulate HIV replication and viral reservoir dynamics^34–37^, providing a platform for studying allo-HSCTs impact on viral reservoirs.

In this study, we modeled CCR5 wild-type allo-HSCTs in four SHIV-infected and ART-suppressed MCMs using our recently established TCRα/β-depleted MHC-matched bone marrow (BM) transplant model, which supports donor chimerism without GvHD^38^. As anticipated, the myeloablative conditioning regimen substantially depleted circulating leukocytes. As a result, we detected approximately 85% whole blood donor chimerism, with three MCMs having undetectable SHIV DNA in their peripheral blood mononuclear cells (PBMCs) post-transplant. However, SHIV-harboring cells persisted in various tissues, indicating that ART-suppression, allo-HSCs, and cytoreductive conditioning alone do not eliminate virally infected cells early after allo-HSCTs. Our findings suggest that extended ART treatments and GvH responses may be needed to eradicate viral reservoirs after allo-HSCTs.

## Results

### SHIV infection and ART suppression

**Figure 1:**
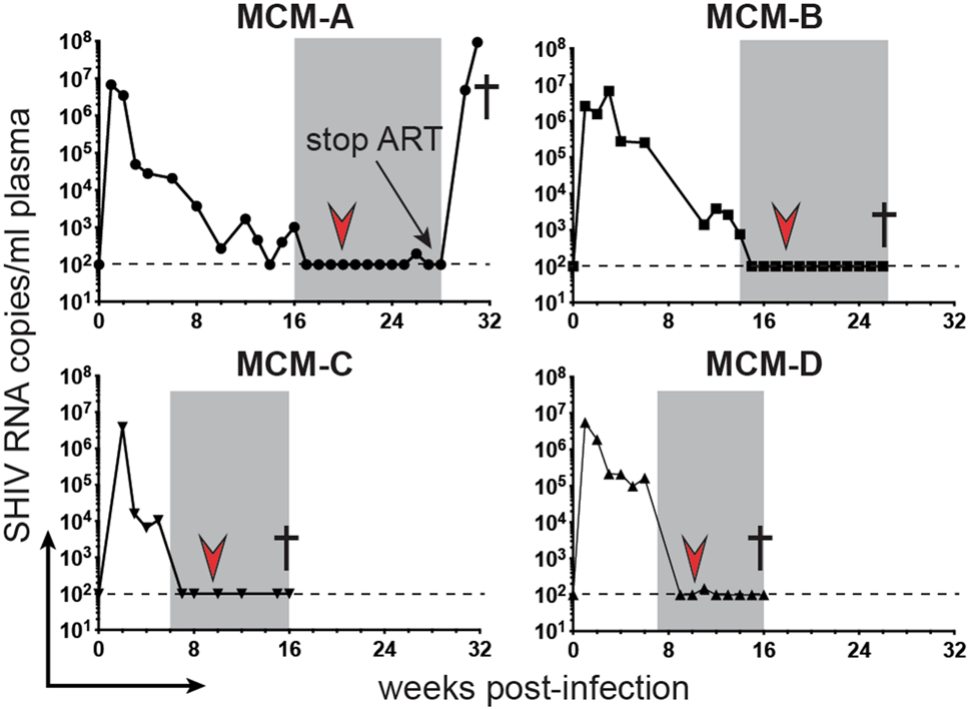
Plasma viral loads after SHIV162P3 infection. Longitudinal plasma viral loads for the SHIV162P3-infected MCMs, with dashed lines indicating the qRT-PCR limit of detection (100 vRNA copy Eq/mL plasma). The gray boxes signify the timing of ART, the red arrows designate allo-HSCs infusions, and † indicate necropsies.

We inoculated four MCMs (MCMs A-D) intravenously with SHIV162P3, a chimeric virus with a CCR5-tropic HIV envelope^39^. As anticipated, virus replication peaked by 3 weeks post-infection (p.i.) and declined thereafter (Fig. 1). To establish robust viral reservoirs, we allowed the SHIV infections to progress untreated for between 6 and 16 weeks p.i. before starting the MCMs on an ART regimen consisting of tenofovir, emtricitabine, and raltegravir. The ART regimen suppressed plasma viremia to undetectable levels within 3 weeks of initiation (< 100 viral RNA (vRNA) copy/eq per mL; Fig. 1). We maintained ART for the duration of the study, except where noted below, keeping plasma viremia undetectable, save for single viral blips in MCMs A and D.

### Allo-HSCT in SHIV-infected, ART-suppressed MCM

Our goal was to model allo-HSCTs with CCR5 wild-type cells in ART-suppressed people with HIV while minimizing the likelihood of severe GvHD. We collected BM aspirates from sex and MHC-matched MCM (Table 1) and depleted cells expressing α/β T-cell receptors (TCRs), removing T cells that are the primary driver of GvHD^38^. Approximately 1 month after starting ART, we delivered myeloablative doses of total body irradiation (TBI; two consecutive days of 5 Gray (Gy), 10 Gy total) and infused the MCMs with α/β T cell-depleted CD34^+^ HSCs (0.6–1.0 × 10^7^/kg; Table 1). Post-transplant, we prophylactically treated the MCMs with cyclophosphamide and tacrolimus to prevent GvHD (Additional file 2: Table S1).

**Table 1:** MCM characteristics and doses of transplanted cells.

Recipient				Donor				Total cells transplanted		CD34^+^ cells		TCRα/β^+^ cells	
ID	MHC type	Sex	Age (years)	ID	MHC type	Sex	Age (years)	Total	Dose (kg)	Total	Dose (kg)	Total	Dose (kg)
MCM-A	M1/M2	F	4	MCM-E	M2/M2	F	9	4.4 × 10^8^	1.5 × 10^8^	2.5 × 10^7^	0.8 × 10^7^	3 × 10^5^	1 × 10^5^
MCM-B	M1/M2	F	4	MCM-F	M1/M2	F	3	8.5 × 10^8^	1.8 × 10^8^	5 × 10^7^	1 × 10^7^	17 × 10^5^	3.6 × 10^5^
MCM-C	M1/M2	F	13	MCM-G	M1/M2	F	11	7.1 × 10^8^	1.2 × 10^8^	4.8 × 10^7^	0.8 × 10^7^	7 × 10^5^	1.1 × 10^5^
MCM-D	M1/M2	M	7	MCM-H	M1/M2	M	6	8.6 × 10^8^	1.6 × 10^8^	3.4 × 10^7^	0.6 × 10^7^	8 × 10^5^	1.4 × 10^5^

**Figure 2:**
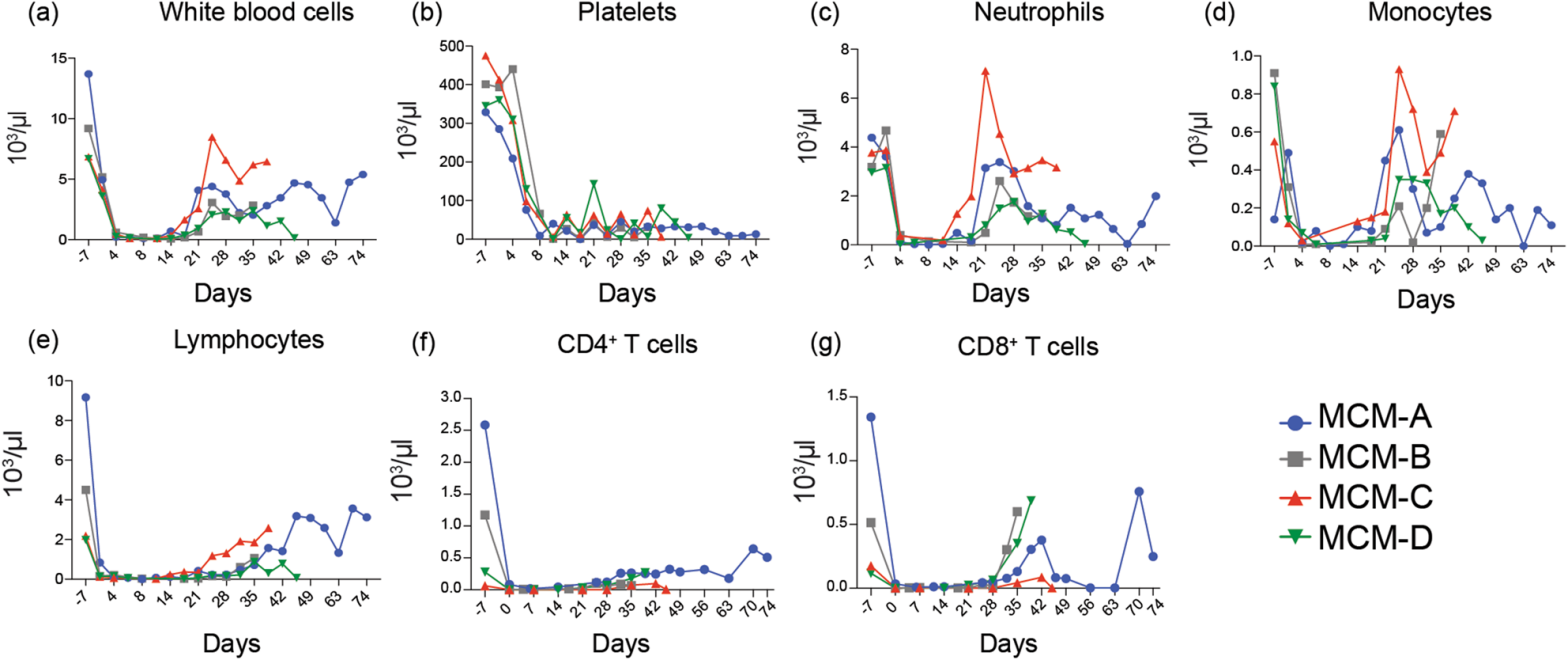
Effect of allo-HSCTs on cell populations and platelets. Longitudinal absolute counts of (**a**) white blood cells, (**b**) platelets, (**c**) neutrophils, (**d**) monocytes, (**e**) lymphocytes, (**f**) CD4^+^ T cells, and (**g**) CD8^+^ T cells after allo-HSCTs. Counts are displayed as × 10^3^/µL.

As expected, the myeloablative conditioning regimen rapidly depleted peripheral white blood cells (WBCs; Fig. 2a). However, the MCMs also developed chronic thrombocytopenia post-transplant (Fig. 2b), a common allo-HSCT complication^40^, which we treated with irradiated whole blood transfusions from SHIV-negative MCMs. Nevertheless, neutrophils reappeared in the peripheral blood of the MCMs by 21 days post-transplant, with absolute counts in MCM-C spiking above pre-transplant levels before rapidly declining (Fig. 2c). The remaining three MCMs had persistent neutropenia after their initial bursts of circulating neutrophils. Likewise, monocytes reemerged in the peripheral blood by 21 days post-transplant, with MCM-C again having the most robust recovery (Fig. 2d). The absolute monocyte counts were more variable than the neutrophil counts post-transplant, gradually declining in MCMs A and D, while rapidly increasing in MCMs B and C before their deaths.

Lymphocytes recovered slowly after the allo-HSCTs (Fig. 2e). In MCM-C, lymphocytes reappeared in the peripheral blood approximately 3 weeks post-transplant and returned to near pre-transplant levels by day 38 post-transplant. Likewise, MCM-A exhibited a slow but steady increase in peripheral lymphocytes, reaching ~ 3500 cells/µL by day 71 post-transplant. In contrast, MCMs B and D had stunted lymphocyte recoveries, with absolute lymphocyte counts failing to reach 1000 cells/µL of blood by 5- and 7-weeks post-transplant, respectively. Similarly, CD4^+^ T cells failed to fully recover in the MCMs blood post-transplant (Fig. 2f). Finally, circulating CD8^+^ T cells increased in MCMs A, B, and D approximately 1-month post-transplant (Fig. 2g), fluctuating in MCM-A until its death. Meanwhile, CD8^+^ T cells poorly recovered in MCM-C, only reaching 86 CD8^+^ T cells/µL blood at 42 days post-transplant.

**Figure 3:**
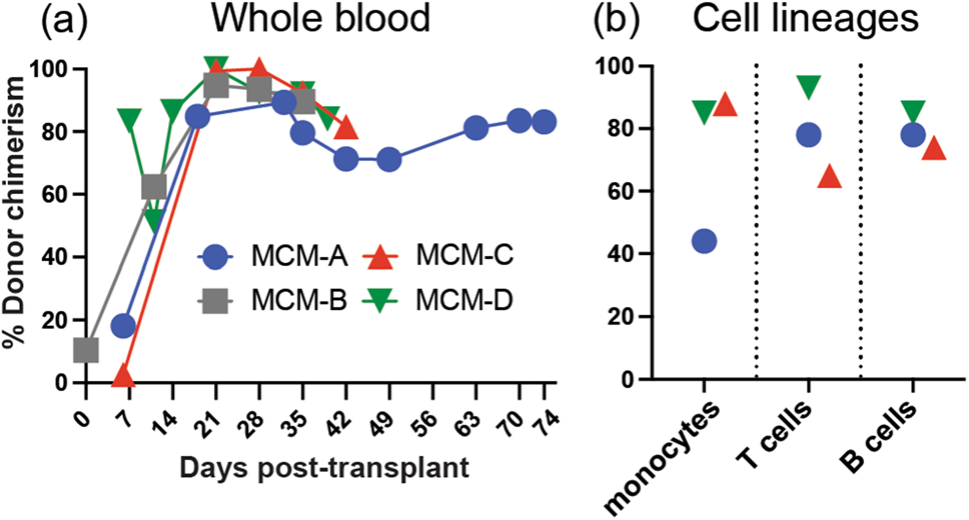
Donor chimerism after allo-HSCT. We determined donor chimerism levels post-transplant by sequencing SNPs differing between the donor and recipient. (**a**) Longitudinal percent donor chimerism in whole blood after allo-HSCT. (**b**) At necropsy, the percent donor chimerism among flow cytometry sorted PBMC monocytes (CD45^mid^CD14^+^ lymphocytes), T cells (CD14^-^CD45^hi^CD3^+^ lymphocytes), and B cells (CD14^-^CD45^hi^CD20^+^).

We measured post-transplant donor engraftment by deep sequencing single-nucleotide polymorphisms (SNPs) unique to the donor and recipient MCMs. Despite low absolute leukocyte counts, the allo-HSCT recipients exhibited greater than 85% whole blood donor chimerism by 3 weeks post-transplant, followed by modest declines to approximately 80% donor chimerism (Fig. 3a). Notably, MCM-A, the longest surviving transplant recipient, maintained 70–90% donor chimerism for 2 months until euthanasia. Additionally, we measured donor chimerism of circulating myeloid (monocytes) and lymphoid (B and T cells) lineages in MCMs A, C, and D at necropsy (cells from MCM-B were unavailable for analysis). The frequencies of donor-derived myeloid and lymphoid cells in MCMs C and D ranged between 65 and 93% of total cells (Fig. 3b), consistent with the whole blood chimerism results (Fig. 3a). In MCM-A, 78% of the B and T cells were of donor origin (Fig. 3b), similar to the 83% of donor cells detected in the whole blood. However, only 45% of the monocytes were of donor origin. Collectively, these results indicate that donor leukocytes replaced recipient cells early after transplant, with notable donor lymphocyte engraftment despite using α/β TCR-depleted grafts.

### Pre- and post-ART viral reservoirs

We designed our GvHD prophylaxis regimen of cyclophosphamide and tacrolimus to minimize high-grade GvHD. As anticipated, GvHD was limited post-transplant with only MCM-A exhibiting transient skin lesions (white flaking/dry skin) that resolved with minimal intervention. Additionally, MCMs C and D had diarrhea, which may have been a TBI side effect. Furthermore, the MCMs had no significant abnormalities in their liver functions or elevated total bilirubin post-transplant, indicating an absence of hepatic GvHD.

**Figure 4:**
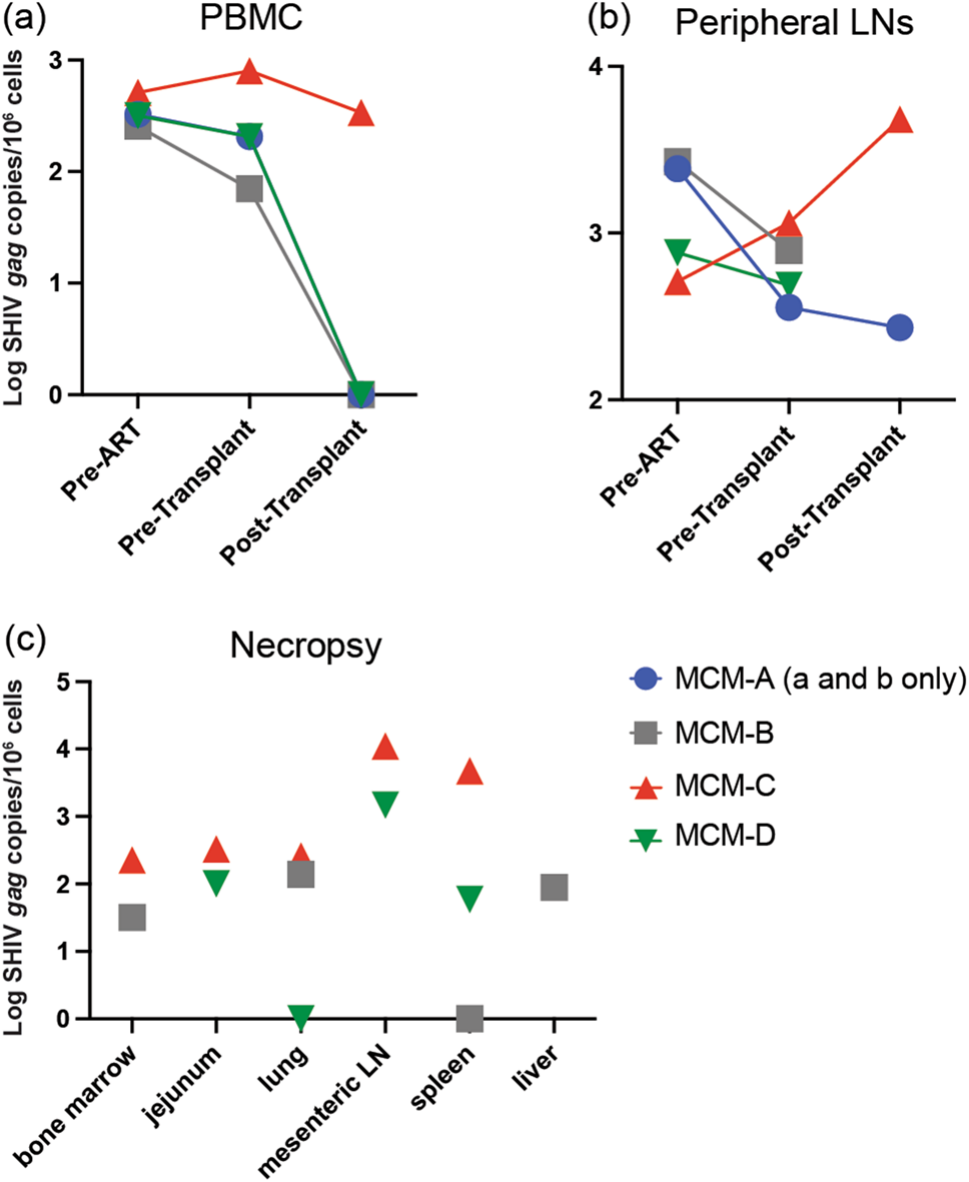
SHIV DNA in allo-HSC transplanted MCMs. We measured copies of vDNA in (**a**) PBMCs and (**b**) peripheral lymph nodes before ART initiation, after ART but before allo-HSCTs, and post-transplant. (**c**) SHIV DNA levels in the tissues of MCMs B, C, and D at necropsy. Total SHIV DNA was measured by digital droplet PCR using SIV *gag*-specific primers and normalized to 10^6^ cells by measuring macaque RNaseP p30, RPP30, in companion ddPCR assays. Post-transplant peripheral lymph node samples from MCM-B and MCM-D were unavailable for analysis.

We measured cell-associated vDNA in PBMCs and inguinal lymph nodes to determine the effect of ART, myeloablative conditioning, and allo-HSCTs on viral reservoirs in the absence of severe GvHD (Fig. 4). Before starting ART, we detected more SHIV-harboring cells in the inguinal lymph nodes (range 743–2680 copy Eq/1 × 10^6^ cells) than the PBMC (range 255–512 copy Eq/1 × 10^6^ cells), consistent with lymph nodes being a primary viral reservoir^41,42^. Additionally, the lymph nodes of MCMs A and B contained more SHIV-infected cells than MCM-D, potentially due to these MCMs starting ART later in infection (14–16 weeks versus 6 weeks). Lastly, the number of SHIV-infected cells declined in the blood of all the MCMs between 36 and 64 days post-transplant, with MCMs A, B, and D having undetectable vDNA in their PBMCs (Fig. 4a).

Nevertheless, virally infected cells persisted in other tissues. MCMs-B, -C, and -D remained on ART until being necropsied, enabling a more thorough examination of post-transplant viral reservoirs. In MCM-C, we detected more vDNA in the inguinal lymph nodes at necropsy than in pre-transplant samples (1151 vs. 4807 vDNA copy Eq/million cells; Fig. 4b). This animal developed viral-induced colitis with ulcers post-transplant, potentially expanding latently infected cells or promoting localized virus replication, spreading SHIV-harboring cells into secondary lymphoid tissues. Nevertheless, we found vDNA outside of the secondary lymphoid tissues in the three MCMs, including the bone marrow, lung, and jejunum (Fig. 4c). Together these results demonstrate that viral reservoirs persist in various tissues early after allo-HSCTs without GvHD.

### SHIV rapidly rebounds after removing ART

ATIs are used in HIV cure studies to evaluate treatment efficacy^43,44^. Therefore, we modeled ATI in MCM-A, an animal with a single viral blip but otherwise suppressed plasma viremia while on ART. One day before stopping ART, day 62 post-transplant, we collected blood and axillary lymph nodes to measure cell-associated SHIV. In these samples, we found no vDNA in the PBMCs but detected 271 vDNA copies/million cells in the lymph nodes, a slight decrease from pre-transplant inguinal lymph nodes (359 vDNA copies/million cells; Fig. 4b). Nevertheless, SHIV rebounded rapidly after removing ART (Fig. 1). Plasma viremia was 1 × 10^7^ vRNA copy eq/mL 7 days after stopping treatment, roughly equivalent to peak acute viremia, and spiked to nearly 1 × 10^8^ vRNA copy eq/mL plasma at 10 days post-ART. These results show that latent reservoirs can reactivate shortly after treatment interruption and initiate viral rebound early after allo-HSCTs.

## Discussion

Allo-HSCT-associated HIV reservoir decay likely results from parallel processes protecting donor cells from infection and eliminating pre-existing latently infected cells^10,11^. Here, we employed the clinically relevant SHIV/MCM model to determine the effect of continuous ART and allo-HSCTs on viral reservoirs in the absence of high-grade GvHD. We recently reported that transplanting MCMs with MHC-matched TCRα/β^+^-depleted HSCs and prophylactically treating them with cyclophosphamide and tacrolimus prevents GvHD post-transplant^38^. Employing this regimen, only one SHIV-infected MCM had transient skin lesions post-transplant. These results contrast with previous NHP allo-HSCT studies where T-cell replete HSCs quickly induced GvHD in ART-suppressed SHIV-infected rhesus macaques^45^ and uninfected MCMs^46^.

Although we prevented GvHD, removing alloreactive TCRα/β^+^ T cells from the bone marrow grafts may have inhibited viral reservoir clearance. Still, our results agree with a previous study transplanting MHC-haploidentical HSCs into ART-suppressed SHIV-infected rhesus macaques that developed GvHD post-transplant^49^. Both studies observed reduced SHIV DNA levels in the blood and lymph nodes post-transplant, with SHIV reservoirs persisting in other tissues. Further, post-transplant follow-up was short in both studies, supporting the need for extended times on ART post-transplant to clear pre-existing latently infected cells.

Supplementing allo-HSCTs with cellular therapies may improve viral reservoir clearance^47,48^. Donor lymphocyte infusions (DLIs) can drive relapsing hematologic tumors into remission^49^. Conceivably, incorporating DLIs into allo-HSCT treatment plans could similarly augment GvVR responses^25^. However, since DLIs involve transferring polyclonal T cells, they also carry a significant risk of GvHD^50^. Therefore, targeted cellular immunotherapies maximizing GvVR effects while minimizing GvHD side effects are preferred. For instance, repurposing cancer adoptive cell therapies may boost GvVR activity^51–53^. HIV-specific chimeric antigen receptor (CAR) T- and NK-cell therapies are promising HIV cure strategies^54^ despite early iterations having limited impact on HIV reservoirs^55–58^. Alternatively, focusing T cells on minor histocompatibility antigens (mHAgs) exclusively expressed by host leukocytes may promote GvVR effects without causing GvHD^59^. We recently identified a mHAg in APOBEC3C that is expressed preferentially by MCM immune cells^60^. Hence, this epitope may serve as a model antigen for testing mHAg-targeted cellular immunotherapies in NHPs and examining their capacity to eradicate viral reservoirs.

In this study, we used the pathogenic SIV-HIV chimera SHIV162P3 to model HIV infections. This recombinant virus contains the *env* gene from the clade B isolate HIV-1 SF162 inserted into the SIVmac239 genomic backbone^61^. Importantly, SHIV162P3 exclusively uses CCR5 as a coreceptor, similar to most transmitted HIV-1 strains^39,62^. This coreceptor usage contrasts with common SIV strains, which can use alternate chemokine receptors and G-protein coupled receptors for cell entry^63,64^. However, SHIV162P3 replication is less robust than most SIV strains, resulting in lower chronic phase viral loads^65,66^. Indeed, MCM-A and -B spontaneously suppressed viremia to approximately 10^3^ vRNA copies/mL plasma by 4 months post-infection. Nevertheless, SHIV162P3 established pre-transplant viral reservoirs in our MCMs consistent with previous SHIV/ SIV studies^41,45,67,68^.

Our study has several limitations. First, its small size and lack of a control group transplanted with TCR α/β-replete HSCs make it difficult to draw firm conclusions from the results. Second, ART was started 3–6 weeks before transplantation. While this provided sufficient time to suppress SHIV replication, it does not reflect the prolonged ART typical of HIV-infected allo-HSCT recipients^4,5,8,14,15^. Third, we could not determine whether virally infected cells were of donor or recipient origin post-transplant. A study by Colonna et al*.* exploited haploidentical allo-HSCTs in rhesus macaques to sort donor and recipient cells based on differential MHC expression, finding that most SHIV-infected cells were from the recipients early after transplantation^45^. However, our MHC-matched MCMs lacked unique surface markers to distinguish donor and recipient cells, prohibiting similar analyses. Lastly, we achieved poor donor cell engraftment, hindering post-transplant analyses and potentially limiting SHIV reservoir depletion. Consequently, we could not quantify donor chimerism in tissues or measure post-transplant antiviral and GvVR immune responses. The limited lymphocyte recovery also hampered our ability to longitudinally measure SHIV-infected cells post-transplant. Therefore, less toxic pre-transplant conditioning regimens, higher HSC doses, or T-cell replete HSCs may be necessary for sustaining donor cell engraftment. To this end, Burwitz et al*.* established an allo-HSCT model in MHC-matched MCMs that achieves durable post-transplant donor chimerism with reduced-intensity conditioning but has an increased risk of GvHD^46^. Thus, allo-HSCTs in NHPs will likely need to balance full donor engraftment with suppressing GvHD to dissect the mechanisms of GvVR effects.

## Conclusions

In summary, MHC-matched bone marrow transplants in ART-suppressed SHIV-infected MCMs reduced, but did not eliminate, viral reservoirs early after transplantation. However, depleting TCRα/β T cells from the HSCs and post-transplant prophylaxis prevented high-grade GvHD. Lastly, our results indicate that this model has room for improvement because the treatment regimen was insufficient for robust donor cell engraftment, limiting lymphocyte recovery and making a detailed analysis of tissues and GvVR immune responses difficult.

## Methods

### Ethics statement and animal care

The staff at the Wisconsin National Primate Research Center (WNPRC) cared for the Cynomolgus macaques (*Macaca fascicularis*) according to the regulations and guidelines of the University of Wisconsin Institutional Animal Care and Use Committee, which approved this study (protocol g005424) following the recommendations of the Weatherall Report and the principles described in the National Research Council's Guide for the Care and Use of Laboratory Animals and in compliance with ARRIVE (Animal Research: Reporting of In Vivo Experiments) guidelines. The MCMs were closely monitored for signs of stress or pain. In consultation with the WNPRC veterinarians, the MCMs were euthanized by anesthetizing with ketamine (≥ 15 mg/kg, intramuscular) followed by intravenous administration of sodium pentobarbital (> 50 mg/kg) when they developed an untreatable opportunistic infection, inappetence, and/or progressive decline in condition.

### MHC typing

MHC genotyping was performed by Genetic Services at WNPRC as previously described^69^. Briefly, genomic DNA (gDNA) was isolated from whole blood or PBMCs and used as templates in polymerase chain reactions (PCRs) with primers flanking exon 2 of MHC class I (Mafa-A, Mafa-B, Mafi-I, and Mafa-E) and class II (Mafa-DRB, Mafa-DQA, Mafa-DQB, Mafa-DPA, and Mafa-DPB) loci. The PCR reactions were generated with a Fluidigm Access Array, which permitted all reactions to be multiplexed in a single experiment. After cleanup with AMPure beads (Beckman Coulter), the amplicons were sequenced on an Illumina MiSeq. The resulting sequences were mapped against a custom database of MCM class I and II sequences to assign one of seven MCM haplotypes (M1-M7).

### SHIV infections and viral loads

We inoculated the MCMs intravenously with 500 median tissue culture infectious doses (TCID_50_) of SHIV162P3^39^. Plasma viral loads were measured via quantitative real-time polymerase chain reactions (qRT-PCRs) as previously described^70,71^. Briefly, vRNA was isolated from plasma using the Maxwell Viral Total Nucleic Acid Purification kit (Promega) on a Maxwell 48 RSC instrument (Promega). Next, vRNA was reverse transcribed into complementary DNA (cDNA), amplified using the TaqMan Fast Virus 1-Step Master Mix qRT-PCR kit (Invitrogen) on a LightCycler 480 or LC96 instrument (Roche), and quantified by interpolation onto a standard curve of tenfold serial dilutions of an SIV *gag *in vitro transcript. The assay limit of detection was 100 vRNA copies/mL.

### Antiretroviral treatment

We treated the SHIV-infected MCMs with a combination of reverse transcriptase inhibitors tenofovir (PMPA) and emtricitabine (FTC) and integrase inhibitor raltegravir (RAL). PMPA and FTC were pre-formulated at concentrations of 20 mg/mL and 40 mg/mL in water containing sodium hydroxide (NaOH) and administered subcutaneously once daily at 1 mL/kg of body weight. 100 mg of RAL was mixed twice daily with food^72^. Gilead Sciences kindly provided PMPA and FTC, and Merck kindly provided RAL through material transfer agreements.

### Donor bone marrow collection and TCRα/β cell depletion

We prepared the HSCs as previously described^38^. Briefly, we collected BM from sex and MHC-matched MCMs (Table 1) by aspirating up to 5 mL from four sites, 20 mL total, and removed red blood cells using ACK lysis buffer (Thermo Fisher Scientific). Next, we washed the cells twice with phosphate-buffered saline and incubated them with anti-TCRα/β allophycocyanin (APC) antibodies (clone R73, BioLegend) for 20 min, followed by incubating with anti-APC microbeads (Miltenyi Biotec) for an additional 20 min at 4 °C. Finally, we passed the stained cells through two LS columns (Miltenyi Biotec) stacked on top of one another, collecting both negative and positive cell fractions, and cryopreserving them at 30 million/mL in serum-free expansion medium (SFEM; Stem Cell Technologies) containing 5% fetal bovine serum and 10% dimethylsulfoxide (DMSO) until further use.

### Bone marrow transplants and care

First, we treated the MCMs with daily oral antibiotics trimethoprim (200 mg) and sulfamethoxazole (40 mg) to decontaminate the gut, starting 7 days pre-transplant (− 7) and continuing until day-1. Next, we started the MCMs on systemic bacterial prophylaxis by administering ceftriaxone (50 mg/kg) on day-2; MCM-D was switched to cefazolin (25 mg/kg twice daily) at day-1, and maintaining treatment until absolute neutrophil counts stabilized. On day-2, the MCMs received five separate 1 Gy fractions of TBI on two consecutive days (10 Gy total), sparing the lungs and eyes as previously described^38^. One day before transplantation, we thawed cryopreserved TCRα/β-depleted BM fractions at 37˚C and cultured them overnight in SFEM containing 100 ng/mL human stem cell factor (SCF, Peprotech), 100 ng/mL human Fms-related tyrosine kinase 3 (FLT3) ligand (Peprotech), and 50 ng/mL human thrombopoietin (TPO, Peprotech). On the day of transplantation, we resuspended the BM cells in 15 mL of Plasma-Lyte (Baxter), a nonpyrogenic isotonic solution, supplemented with 2% autologous serum and 5 U/mL heparin, and infused the cell suspension intravenously. Next, we prophylactically treated the MCMs for GvHD using cyclophosphamide (50 mg/kg) on days 4 and 5 post-transplant, followed by twice-daily tacrolimus (0.01 mg/kg) starting at day 5, maintaining tacrolimus serum levels between 5 and 15 ng/mL. To prevent fungal infections, we treated the MCMs with once-daily fluconazole (5 mg/kg) starting on day 0. Lastly, we treated MCMs C and D with oral eltrombopag (1.5 mg/kg) and N-acetyl l-cysteine (50 mg/kg), respectively, to support platelet engraftment. See Additional file 2: Table S1 for treatment details.

### Measuring peripheral leukocyte populations and GvHD

Following transplantation, we collected peripheral blood from the MCMs twice per week for the first month, followed by bi-weekly blood draws for the next 2 months and monthly thereafter. We used an XS-1000i automated hematology analyzer (Sysmex) to measure platelets and leukocyte populations. In addition, GvHD was evaluated as previously described^73^.

We quantified CD4^+^ and CD8^+^ T cells by incubating 100 µL of whole blood with anti-CD8-Brilliant Violet 421 (clone SK1; BioLegend), anti-CD4-APC (clone L200; BD-Pharmingen), and anti-CD3-Alexa 700 (clone SP34; BD-Pharmingen) antibodies with Live/Dead Fixable Near-IR vital dye (ThermoFisher Scientific) for 30 min at 4˚C. Next, we lysed the red blood cells by adding 1 mL of FACS Lysing Solution (BD Biosciences) and incubated the samples for 10 min at room temperature. We washed the cells three times with FACS buffer (phosphate-buffered saline supplemented with 2% fetal calf serum), fixed with 2% paraformaldehyde, and ran the samples on a BD-LSR-II flow cytometer using FACSDiva software (Becton Dickinson). Finally, we analyzed the flow cytometry data using FlowJo software (Treestar), determining the absolute CD4^+^ and CD8^+^ T cell counts by multiplying the frequency of CD4^+^ or CD8^+^ T cells (singlets, live, CD3^+^, CD4^+^ or CD8^+^) by the white blood counts per microliter of blood from the matching complete blood counts (Additional file 1: Figure S1).

### Lymphocyte isolation from tissues

We isolated lymphocytes from the blood, lymph nodes, spleen, and bone marrow as previously described^74^. Briefly, we isolated PBMCs from EDTA-anticoagulated blood by density gradient centrifugation using Ficoll-Paque PLUS (GE Healthcare), removing contaminating red blood cells with ACK lysis buffer. We collected peripheral lymph nodes before ART (MCM A and B: 77 days post-infection (dpi); MCM-C: unavailable; and MCM-D: 34 dpi), after ART but before allo-HSCT (MCM-A: 23 days post-ART; MCM-B: 24 days post-ART; MCM-C: 18 days post-ART; and MCM-D: 12 days post-ART). Additionally, we collected lymph nodes from MCM-A 62 days post-transplant but before ART cessation and the necropsies for MCM C and D, which occurred on days 46 and 39 post-transplant, respectively, while they were on ART. We were unable to collect lymph nodes at MCM-B's necropsy for analysis.

We isolated lymphocytes from the lymph nodes, spleen, and bone marrow by dicing the tissues with scalpels and forcing the cells through 100-µm cell strainers to remove connective tissues. Lymphocytes were enriched from the cell suspensions using Ficoll-Paque PLUS density gradient centrifugation.

### Measuring donor chimerism

We identified SNPs that distinguished the donor and recipient MCMs using a panel of 12 SNPs (see Additional file 3:Table S2) and the rhAMP SNP Genotyping System (Integrated DNA Technologies) as described previously^38,75^, giving preference to homozygous/homozygous mismatches. We performed the rhAMP assays in triplicate using a LightCycler 96 Instrument (Roche Molecular Systems) in the endpoint analysis mode.

We quantified donor chimerism in whole PBMCs or immune subsets by sequencing the diagnostic SNPs with an Illumina MiSeq using previously described primer sets, PCR conditions, and analysis pipeline (see also Additional file 4:Table S3 and Additional file 5:Table S4)^38^. To isolate specific immune subsets, we stained PBMCs with anti-CD3 FITC (clone SP34; BD Pharmingen), anti-CD14 PerCP-Cy5.5 (clone M5E2; BioLegend), anti-CD20 APC (clone 2H7; BioLegend), anti-CD45 PE (clone D058-1283; BD Pharmingen), and Live/Dead Fixable Near-IR vital dye (ThermoFisher Scientific) and sorted into monocyte (singlets, live, lymphocytes, CD45^mid^, CD14^+^ cells), T lymphocyte (singlets, live, lymphocytes, CD14^−^, CD45^hi^, CD3^+^), or B lymphocyte (singlets, live, lymphocytes, CD14^−^, CD45^hi^, CD20^+^) populations using a BD FACSJazz cell sorter. We used between 179 and 61,251 cells for gDNA extraction and sequencing for each sorted population.

### Quantifying cell-associated viral DNA

We extracted gDNA from tissues or PBMCs using the DNeasy Blood & Tissue Kit (Qiagen) per the manufacturer's instructions, except eluting the DNA twice with separate 75 µL of molecular grade water (150 µL total volume). We determined the concentration of DNA using a Nanodrop spectrophotometer (ThermoFisher Scientific), and we digested up to 3 µg of DNA with 3 µL of EcoRI-HF restriction enzyme (New England Biolabs) per 50 µL.

We performed two digital droplet PCR (ddPCR) reactions for each sample: one quantifying vDNA and the other determining total cell number. First, we quantified vDNA using the primers and probes for SIV *gag* and the cycling conditions described by Gama et al*.*^76^. Second, we normalized vDNA copies to cell numbers by quantifying the RNase P (RPP30) gene, as previously described^77^. We generated droplets and read the samples on a QX200 ddPCR system (Bio-Rad). We quantified the target genes in duplicate for each sample across at least two independent runs.

## Supplementary Information

Supplementary Figure S1.

Supplementary Table S1.

Supplementary Table S2.

Supplementary Table S3.

Supplementary Table S4.

## Data Availability

The data that support the findings of this study are available within the paper and Supplementary files. The datasets generated during the study are available from the corresponding author upon reasonable request.

## Abbreviations

Allo-HSCTs: Allogeneic hematopoietic stem cell transplants
ART: Antiretroviral therapy
HIV: Human immunodeficiency virus
SHIV: Simian-human immunodeficiency virus
MCMs: Mauritian cynomolgus macaques
GvH: Graft-versus-host
GvHD: Graft-versus-host disease
GvVR: Graft-versus-viral reservoir
CCR5: C-C chemokine receptor type 5
CXCR4: C-X-C chemokine receptor type 4
CCR5∆32: C-C chemokine receptor type 5 with 32 base-pair deletion
MHC: Major histocompatibility complex
NHPs: Nonhuman primates
BM: Bone marrow
p.i.: Post-infection
Gy: Gray
WBCs: White blood cells
ATIs: Analytical treatment interruptions
DLIs: Donor lymphocyte infusions
CAR: Chimeric antigen receptor
mHAgs: Minor histocompatibility antigens
WNPRC: Wisconsin national primate research center
gDNA: Genomic deoxyribonucleic acid
PCRs: Polymerase chain reactions
Mafa: *Macaca fascicularis*
TCID_50_: Median tissue culture infectious doses
qRT-PCRs: Quantitative real-time polymerase chain reactions
cDNA: Complementary deoxyribonucleic acid
PMPA: Tenofovir (9-[9(R)-2-(phosphonomethoxy)propyl]adenine
FTC: Emtricitabine
RAL: Raltegravir
SFEM: Serum-free expansion medium
TBI: Total body irradiation
SCF: Stem cell factor
FLT3: Fms-related tyrosine kinase 3
TPO: Thrombopoietin
PBMCs: Peripheral blood mononuclear cells
SNPs: Single-nucleotide polymorphisms
vDNA: Viral deoxyribonucleic acids
ddPCR: Digital droplet polymerase chain reaction
RPP30: RNase P subunit p30
