# Supplementary material for: Allogeneic MHC-matched T-cell receptor α/β-depleted bone marrow transplants in SHIV-infected, ART-suppressed Mauritian cynomolgus macaques
Source: Sci Rep. 2022 Jul 19;12:12345. doi: 10.1038/s41598-022-16306-z (PMC9296477; doi:10.1038/s41598-022-16306-z)
Supplement: Supplementary file 1 — Supplementary Figure S1. [file 41598_2022_16306_MOESM1_ESM.pdf]

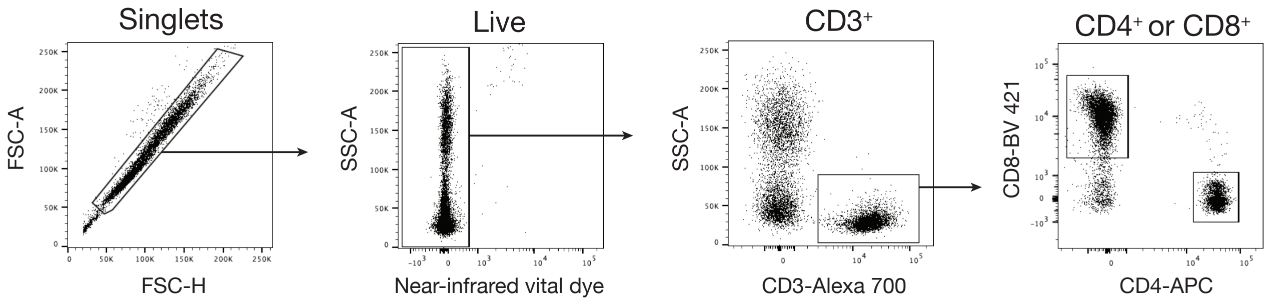

Supplementary figure 1. Flow cytometric gating strategy for determining the frequency of $CD4^+$ and $CD8^+$ T cells in the blood, progressively selecting singlets, live cells, $CD3^+$, and either $CD4^+$ or $CD8^+$ cells. Representative data from a pre-transplant sample.
