# Supplementary material for: Allogeneic MHC-matched T-cell receptor α/β-depleted bone marrow transplants in SHIV-infected, ART-suppressed Mauritian cynomolgus macaques
Source: Sci Rep. 2022 Jul 19;12:12345. doi: 10.1038/s41598-022-16306-z (PMC9296477; doi:10.1038/s41598-022-16306-z)
Supplement: Supplementary file 2 — Supplementary Table S1. [file 41598_2022_16306_MOESM2_ESM.pdf]

Supplemental Table 1. Transplant recipients' treatment regimens.

MCM-A

days post-transplant

Treatment	Purpose	Dose	Route	-30	..	- 7	..	-2	-1	0	1	2	3	4	5	..	9	..	19	..	26	..	42	..	58	..	62	63	..	74
ART- TDF FTC	Anti-retroviral	20mg/kg 40mg/kg	SQ SQ																											
ART- RAL	Anti-retroviral	100mg	oral																											
Trimethoprim/ sulfamethoxazole	Gut decontamination	200mg 40mg	oral																											
Ceftriaxone	Antibiotic	50 mg/kg	IM																											
Total body irradiation	Immune conditioning	5Gy for 2 days	-																											
TCRα/β-depleted HSCs		2x10^8 cells	IV																											
Cyclophosphamide	Immune suppressant	50 mg/kg	IV																											
Tacrolimus	Immune suppressant	0.01 mg/kg	IM																											
Fluconazole	Anti-fungal	5 mg/kg	oral																											
Blood transfusion			IV																											
Necropsy																														

MCM-B

days post-transplant

Treatment	Purpose	Dose	Route	-42	..	- 7	..	-2	-1	0	1	2	3	4	5	..	12	..	19	..	26	..	33	..	38
ART- TDF FTC	Anti-retroviral	20mg/kg 40mg/kg	SQ SQ																						
ART- RAL	Anti-retroviral	100mg	oral																						
Trimethoprim/ sulfamethoxazole	Gut decontamination	200mg 40mg	oral																						
Ceftriaxone	Antibiotic	50 mg/kg	IM																						
Total body irradiation	Immune conditioning	5Gy for 2 days	-																						
TCRα/β-depleted cells		8.5x10^8 cells	IV																						
Cyclophosphamide	Immune suppressant	50 mg/kg	IV																						
Tacrolimus	Immune suppressant	0.01 mg/kg	IM																						
Fluconazole	Anti-fungal	5 mg/kg	oral																						
Blood transfusion			IV																						
Necropsy																									

MCM-C

days post-transplant

Treatment	Purpose	Dose	Route	-24	..	- 7	..	-2	-1	0	1	2	3	4	5	..	11	..	19	..	28	..	34	35	..	37	..	42	..	45
ART- TDF FTC	Anti-retroviral	20mg/kg 40mg/kg	SQ SQ																											
ART-RAL	Anti-retroviral	100mg	oral																											
Trimethoprim/ sulfamethoxazole	Gut decontamination	200mg 40mg	oral																											
Ceftriaxone	Antibiotic	50 mg/kg	IM																											
Total body irradiation	Immune conditioning	5Gy for 2 days	-																											
TCRα/β-depleted cells		7.1x10^8 cells	IV																											
Cyclophosphamide	Immune suppressant	50 mg/kg	IV																											
Tacrolimus	Immune suppressant	0.01 mg/kg	IM																											
Fluconazole	Anti-fungal	5 mg/kg	oral																											
Blood transfusion			IV																											
Eltrombopag	Support platelet engraftment	1.5 mg/kg	oral																											
Necropsy																														

MCM-D

days post-transplant

Treatment	Purpose	Dose	Route	-20	..	-14	..	- 7	..	-2	-1	0	1	2	3	4	5	..	11	..	18	..	25	..	33	..	39
ART- TDF FTC	Anti-retroviral	20mg/kg 40mg/kg	SQ SQ																								
ART-RAL	Anti-retroviral	100mg	oral																								
Trimethoprim/ sulfamethoxazole	Gut decontamination	200mg 40mg	oral																								
Ceftriaxone	Antibiotic	50 mg/kg	IM																								
Cefazolin	Antibiotic	330 mg/ml	IM																								
Total body irradiation	Immune conditioning	5Gy for 2 days	-																								
TCRα/β-depleted cells		8.6x10^8 cells	IV																								
Cyclophosphamide	Immune suppressant	50 mg/kg	IV																								
Tacrolimus	Immune suppressant	0.01 mg/kg	IM																								
Fluconazole	Anti-fungal	5 mg/kg	oral																								
Blood transfusion			IV																								
N-acetyl L-cysteine	Support platelet engraftment	50mg/kg	oral																								
Necropsy																											
