# Supplementary material for: Allogeneic MHC-matched T-cell receptor α/β-depleted bone marrow transplants in SHIV-infected, ART-suppressed Mauritian cynomolgus macaques
Source: Sci Rep. 2022 Jul 19;12:12345. doi: 10.1038/s41598-022-16306-z (PMC9296477; doi:10.1038/s41598-022-16306-z)
Supplement: Supplementary file 3 — Supplementary Table S2. [file 41598_2022_16306_MOESM3_ESM.pdf]

Supplementary Table 2. Panel of single nucleotide polymorphisms (SNPs) and primers used to identify diagnostic SNPs that distinguish donor and recipient MCMs.

Gene	Chromosome	Position	Allele Change	Allele Specific Primers	Locus Specific Primer
CELSR2	1	112321563	C → T	CCCTGCAGTGAATTACGA[C/T]	GCGCTTCCTGCCCTCATC
GIPR	19	52125587	C → T	ATGGTCATGAGGATGGG[G/A]	GCGAGCGCAACGAAGTCAA
GPR107	15	8694018	C → G	CCTCAGGGTCGGCT[C/G]	GCTCCTTGTTCAGTGTGACAACAT
GPR139	20	19030347	A → G	ATATCGCTGTCTGCCA[T/C]	GCGTGCTGATGTAGTCTTCAGTC
GPR149	2	133230027	A → T	CGCTTTGATCCTAGCACTTAC[A/T]	GCCTTGTGAGCAACTGCACTTTTA
GPR183	17	79668813	G → A	GCCTCTGCATTACAGCCT[C/T]	GCATGACAACCAAGGCTAGTAAGTTT
GPR68	7	154450353	G → T	TGATGTAGATGTTCTCGTAGAG[G/T]	GCGCGTGTACCTGTGCAA
GPR98	6	86863653	C → T	TATGGAAAACCAGAAGATTGAAAG[C/T]	GCCTCACATCTCCTTTAGTCCCT
HTR5A	3	192188304	A → G	GGTCAACTATTGGGACATAC[T/C]	GCGGATAGGAGACCATAGTTCCA
MC4R	18	53659440	G → A	GATTGCTGTCCTCCC[C/T]	GCCCAATCAGGATGGTCAAAGTAAT
P2RY11	19	9916169	G → A	AAGCTGCGTGTGGC[G/A]	GCCCGAGCATCCACGTTGA
TAAR1	4	131253869	C → T	ATCTCTTCAGCGCCTTTGAA[G/A]	GCTGTCTTTCATCTCCATTGACCG

We genotyped each donor/recipient pair using the rhAMP SNP Assay (IDT) to identify diagnostic SNPs, with preference given to homozygous/homozygous mismatches. The chromosome positions are relative to the rhesus macaque genome assembly rheMac2.
