# Supplementary material for: Allogeneic MHC-matched T-cell receptor α/β-depleted bone marrow transplants in SHIV-infected, ART-suppressed Mauritian cynomolgus macaques
Source: Sci Rep. 2022 Jul 19;12:12345. doi: 10.1038/s41598-022-16306-z (PMC9296477; doi:10.1038/s41598-022-16306-z)
Supplement: Supplementary file 4 — Supplementary Table S3. [file 41598_2022_16306_MOESM4_ESM.pdf]

Supplementary Table 3. SNPs used to distinguish donor and recipient MCMs.

Donor	Recipient	SNP	Donor/Recipient Expected	Donor Basepair	Recipient Basepair
MCM-A	MCM-E	CELSR2	Homozygous/Homozygous	C	T
MCM-B	MCM-F	CELSR2	Homozygous/Heterozygous	C	C / T
		MC4R	Heterozygous/Homozygous	C / T	T
MCM-C	MCM-G	MC4R	Homozygous/Homozygous	C	T
MCM-D	MCM-H	HTR5A	Heterozygous/Homozygous	G / A	G
		GPR183	Homozygous/Heterozygous	T	C / T
