# Supplementary material for: Allogeneic MHC-matched T-cell receptor α/β-depleted bone marrow transplants in SHIV-infected, ART-suppressed Mauritian cynomolgus macaques
Source: Sci Rep. 2022 Jul 19;12:12345. doi: 10.1038/s41598-022-16306-z (PMC9296477; doi:10.1038/s41598-022-16306-z)
Supplement: Supplementary file 5 — Supplementary Table S4. [file 41598_2022_16306_MOESM5_ESM.pdf]

Supplementary Table 4. Sequencing primers for amplifying diagnostic SNPs.

Illumina Sequencing Primers

Gene	5' Primer	3' Primer	Amplicon Length (bp)
CELSR2	GGAGCTGCTCCTGGGTGAC	CAACACCTACCAAAGGAGCCTT	150
GPR183	CACCTCCTCAGGGAAATGA	GAACAATGACAACCAAGGC	132
HTR5A	GAGACCATAGTTCCAGGCT	TCCCTGCTTTCATGGATAGGA	165
MC4R	TTGGCTCTCATGGCTTCTCTC	CAGCAGACAACAAAGACGCC	161
